# Unveiling the Silent Regulators: Noncoding RNAs in Parasitic Disease Diagnosis and Therapy

**DOI:** 10.1155/japr/4071834

**Published:** 2026-06-10

**Authors:** Neyon Loku Gamage, Koshila Ranasinghe

**Affiliations:** ^1^ Department of Zoology and Environmental Management, Faculty of Science, University of Kelaniya, Kelaniya, Sri Lanka, kln.ac.lk

**Keywords:** biomarkers, gene silencing, host–pathogen interactions, lncRNA, miRNA, parasitic diseases, siRNA

## Abstract

Parasitic diseases remain a major global health burden, particularly in tropical and low‐resource regions, where limitations in early diagnosis and effective therapeutics contribute to high morbidity and mortality. Emerging evidence highlights noncoding RNAs (ncRNAs) as critical regulators of host–parasite interactions, offering novel opportunities for diagnosis, prognosis, and targeted therapy. However, most of these findings are derived from in vitro experiments and preclinical models with limited validation in clinical settings. This review comprehensively examines the roles of microRNAs (miRNAs), small interfering RNAs (siRNAs), and long noncoding RNAs (lncRNAs) in the diagnosis and treatment of major parasitic diseases, including malaria, leishmaniasis, trypanosomiasis, Chagas disease, helminthic infections, cryptosporidiosis, and scabies. Distinct miRNA expression profiles are associated with disease presence, severity, and clinical outcomes, positioning them as promising noninvasive biomarkers detectable in body fluids. Specific miRNAs such as miR‐223, miR‐155, miR‐145, and miR‐3158 modulate immune responses, inflammation, and parasite survival across multiple infections. Beyond diagnostics, miRNAs actively participate in disease pathogenesis and host defense, enabling therapeutic modulation of immune pathways. siRNA‐based approaches further demonstrate therapeutic potential through RNA interference–mediated silencing of essential parasite genes, successfully impairing parasite growth, viability, and development in diseases such as cystic echinococcosis, cryptosporidiosis, and scabies. Additionally, lncRNAs have emerged as key regulators of innate and adaptive immunity, influencing cytokine signaling, macrophage differentiation, and inflammatory pathways while serving as novel biomarkers for parasitic cardiomyopathies and protozoan infections. Despite promising advances, the clinical translation of ncRNA‐based diagnostics and therapeutics faces significant challenges, including delivery efficiency, stability, specificity, off‐target effects, and ethical considerations. Continued research, improved delivery platforms, and well‐designed clinical trials are essential to harness the full potential of ncRNAs. Therefore, while ncRNA‐based strategies represent a promising direction for future research, their application in parasitic disease diagnosis and therapy remains largely at an experimental level. Continued investigation of ncRNA‐based methods is required to address current limitations and enable safe and effective clinical translation.

## 1. The Silent Regulators: Noncoding RNAs (ncRNAs)

ncRNAs are RNAs that do not encode a protein, but this does not imply that this kind of RNA does not carry information or have functions [1]. It has been assumed that most genetic information is transacted by proteins, but recent evidence suggests that most of the genomes of complex organisms are transcribed into ncRNAs [1]. The human genome consists of about 1%–2% coding sequences, whereas about 98%–99% are noncoding. There are two main types of ncRNAs: housekeeping ncRNAs and regulatory RNAs. Under housekeeping ncRNAs, there are four types of RNAs: rRNA, tRNA, snRNA, and snoRNA. Regulatory RNAs can also be divided into short ncRNAs and long ncRNAs (lncRNAs).

Most ncRNAs are important in many generic functions, such as rRNAs and tRNAs, which help in mRNA translation, whereas small nuclear RNAs are involved in mRNA splicing mechanisms. Modification of rRNAs is done by small nucleolar RNAs [1]. There are three types of small ncRNAs: microRNAs (miRNAs), small interfering RNAs (siRNAs), and Piwi‐interacting RNAs (piRNAs) [2]. lncRNAs are also of three types: long intergenic ncRNAs (lincRNAs), enhancer RNAs (eRNAs), and antisense RNAs [3].

### 1.1. miRNAs

There are at least 700 miRNAs in the human body, and many studies have indicated that miRNAs can be readily detected in many multicellular organisms [2]. miRNAs are short regulatory RNAs involved in post‐transcriptional gene regulation [4]. They also adjust modifications of histones and DNA methylation. Around 30% of the protein‐coding genes in the human body are controlled by miRNAs [5]. During miRNA biogenesis, most miRNAs are transcribed with RNA polymerase II, and they have a poly(A) tail with a 5 ^′^ cap [6]. One or many hairpins characterize the transcription of primary miRNA, which also enclose the functional mature miRNA inside their stems [4].

### 1.2. siRNAs

siRNAs are one of the modes of RNA interference (RNAi). RNAi is a natural defense mechanism against the invasion of exogenous genes [7]. siRNAs are short duplexes that are cut from long stretches of double‐stranded RNA (dsRNA) using an enzyme called Dicer inside the animal body. siRNA has prefect matches with the targeted RNA, which is directed for degradation [8]. Through different mechanisms inside both animals and plants, dsRNA can produce siRNA, which can silence transposons and also participate in the regulatory pathway of controlling gene expression during development [9].

### 1.3. lincRNAs

These lincRNAs consist of more than 200 nucleotides [10]. As lincRNAs have longer lengths, they can fold on themselves, providing a second layer of functionality; because of that, they can recognize targets by base pairing and also through tertiary structural defined surface interactions [11]. They can associate physically with chromatin regulatory proteins and also with their promoters, which are considered to be the target sites for transcription factors. These lincRNAs have a major effect on gene expression patterns [12]. Biological processes such as cell proliferation, morphogenesis, pluripotency, development, neuronal processes, and gametogenesis are some areas where lincRNAs were found [13]. In parasitic infections, lncRNAs are important as key regulators of host immune responses and interactions between host and parasite. They modulate immune responses while interacting with DNA, RNA, and proteins in order to control gene expression. They can influence the activity of transcription factors and chromatin‐ modifying complexes by acting as molecular scaffolds, guides, or decoys. These methods are allowing lncRNAs to control the expression of genes related to immunological signaling pathways such as inflammatory responses and cytokine synthesis [14].

After ncRNAs are categorized into miRNAs, siRNAs, and lincRNAs, it is important to contextualize their functional significance and available data on parasitic diseases (Table 1). The well‐established functions of miRNAs and siRNAs in post‐transcriptional gene regulation and RNAi pathways, which are conserved across several biological systems, are examples of evidence‐based findings [16]. The role of ncRNAs in regulating host immune responses and parasite gene expression in model systems is further supported by preclinical research. Most ncRNA‐based treatments are still at the experimental level because of the lack of functional validation and clinical research, especially those involving lincRNAs and cross‐species regulatory interactions [17].

**Table 1:** Different types of noncoding RNA found in the human genome.

ncRNA		Types	Size	Main functions	References
	Long ncRNA (> 200 nt)	circRNA	100~100 s	miRNA sponge.	[15]
		Intronic lncRNA	10~1000 s	Heterogeneous functions in a wide range of biological processes.	
		lincRNA			
		Sense lncRNA			
	Small ncRNA (< 200 nt)	snoRNA	60–300	Component of nuclear ribonucleoprotein particle (snoRNPs) and guide snoRNP to chemically modify pre‐rRNA to form mature rRNA.	
		snRNA	~150	Component of small nuclear ribonucleoprotein particles (snRNA) and involved in RNA splicing.	
		miRNA	~22	Operate in the RNA interference (RNAi) pathway, bind to target mRNA, and mediate mRNA degradation or translation inhibition.	
		siRNA	20–25	Similar to miRNA, operate in the RNA interference (RNAi) pathway, bind to target mRNA, and mediate mRNA degradation or translation inhibition.	
		piRNA	24–32	Associate with piwi proteins involved in epigenetic and post‐transcriptional silencing of transposons.	

Building on this, both host‐derived and parasite‐derived processes are involved in ncRNA‐mediated regulation of parasitic illnesses. These mechanisms differ greatly in their biological roles and therapeutic implications. While host‐derived ncRNAs, particularly miRNAs, are crucial in influencing immune responses, inflammation, and cellular signaling pathways during infection, parasite‐derived ncRNAs mainly control intrinsic processes like development, metabolism, and immune evasion [18].

Despite these developments, the poor conservation of ncRNA targets among parasite species limits the wider use of ncRNA‐based strategies. Many ncRNA sequences and their regulatory interactions are extremely species specific due to extensive genetic diversity and evolutionary divergence, which limits the applicability of findings from well‐studied systems like *Plasmodium* spp. to other parasitic organisms [19]. The majority of ncRNA‐mediated interactions require species‐specific confirmation, despite the existence of some shared pathways, especially in basic cellular activities. This heterogeneity must therefore be taken into consideration when developing ncRNA‐based treatments, underscoring the need for comparative genomic and transcriptome analyses to identify conserved regulatory regions and enhance cross‐species applicability.

**Figure 1:**
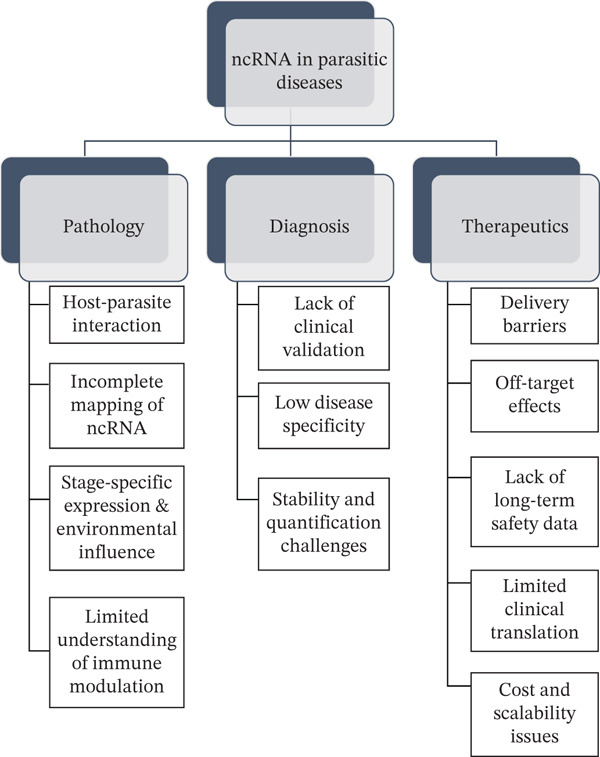
Key knowledge gaps in the application of ncRNAs in parasitic diseases. (Author‐made).

Instead of growing interest in ncRNA‐based approaches, their applications in parasitic diseases remain unclear, while making multiple knowledge gaps across pathology, diagnosis, and therapeutics. Figure 1 highlights those limitations and information that will be discussed in this review.

## 2. ncRNA as a Tool for Parasitic Diseases Diagnosis and Therapeutics

**Figure 2:**
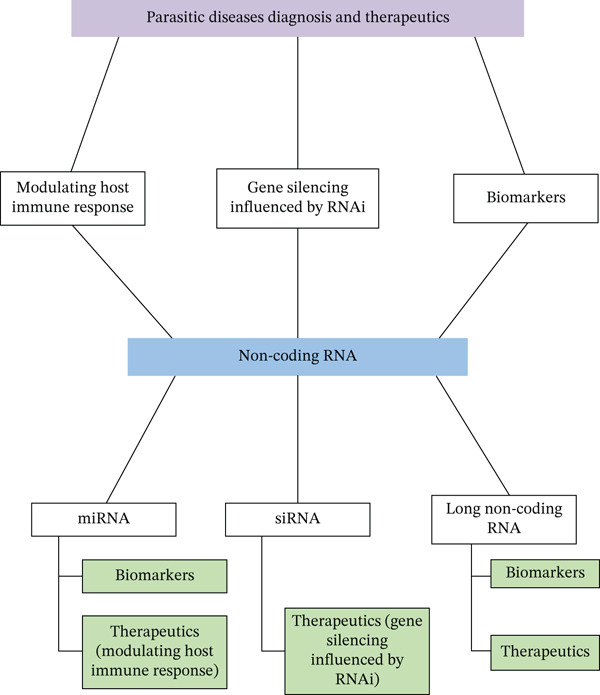
Mechanism of noncoding RNAs in parasitic disease diagnosis and therapeutics (Author‐made).

Parasitic diseases are infectious diseases that are caused by parasites that infect the body. The parasites could be protozoans, worms, nematodes, amoeba, helminthic parasites, arthropod parasites, etc. [20]. The ncRNAs are a diverse group of RNA molecules that do not code for proteins but can be used to diagnose and treat parasitic diseases (Figure 2). Pathogens show a wide range of techniques to invade, replicate, and survive within the host body. These are the places where ncRNAs play a major role while activating intracellular signaling pathways and transcription factors [21]. The reprogramming of the cellular transcripts allows immune‐associated genes to express and modulate [22].

There is a lot of research being done on the possible uses of ncRNAs in parasitic diseases, especially in the field of diagnosis and treatment. While techniques like RNAi provide chances for targeted gene silencing in parasites, circulating ncRNAs have been suggested as noninvasive indicators that can be used for the early detection of diseases [23, 24]. Nevertheless, the majority of ncRNA‐based applications are still in the experimental or preclinical stages. Therefore, it is important to differentiate between the knowledge that is already established and the knowledge that is still speculative in nature, while discussing the role of ncRNAs in parasitic diseases. Even though the potential of the ncRNA‐based approach is high, its application is yet to be validated [24].

## 3. Delivery Strategies for ncRNA Therapeutics

The therapeutic applications of ncRNAs, including miRNAs, siRNAs, and lincRNAs, are fundamentally dependent on safe delivery and an efficient system [24]. Despite significant preclinical promise, delivery‐related issues like instability in biological fluids, quick nuclease degradation, off‐target bio distribution, and ineffective cellular uptake continue to be the main obstacles to the clinical translation of ncRNA‐based therapies [25, 26]. There are mainly three types of delivery mechanisms, such as lipid nanoparticles (LNPs), viral vector‐based delivery, and ligand‐conjugated and targeted delivery systems.

Currently, the most sophisticated and clinically validated ncRNA delivery method is LNPs. LNPs promote effective intracellular release of RNA molecules, increase the cellular uptake via endocytosis, and protect ncRNA delivery from enzymatic destruction [25, 27, 28]. Strong proof of concepts for translational applicability is provided by their effectiveness in RNA treatments, particularly siRNA‐based treatments [24]. In preclinical models, LNPs have shown effective gene silencing, immunological regulation, and tissue‐specific delivery in preclinical models, especially in hepatocytes and phagocytic immune cells, which are important targets in leishmaniasis and malaria‐like parasitic diseases [26, 28]. Nevertheless, several limitations still exist, including inefficient endosomal escape, partly off‐target bio distribution, and possible toxicity related to lipid components [24, 25]. Other than that, these LNP formulations have to be stored in a cold chain frequently, which limits their use in tropical and resource‐constrained environments [28]. Despite these difficulties, LNPs continue to be the most balanced approach in terms of clinical suitability, scalability, and delivery efficiency.

Among the viral‐vector delivery methods, adenoviral, lentivirus, and adeno‐associated viral (AAV) systems are examples of viral vectors that offer extremely effective transport and long‐term expression of ncRNA methods [29, 30]. Strong gene transfer and long‐term expression have been shown in preclinical research, which could be helpful for persistent parasite infections that need ongoing gene regulation [29]. However, safety concerns such as immunogenicity, insertional mutagenesis, and possible activation of host immune responses can limit the use of these delivery mechanisms [30]. Because of the high cost of manufacture, stringent biosafety regulations, and intricate regulatory processes, especially in low‐ and middle‐income countries (LMICs), the use of viral‐vector delivery methods for parasitic infections is limited [29].

Ligand‐conjugated delivery mechanisms, including antibody‐peptide and aptamer‐based platforms, provide improved target specificity [24, 26]. These mechanisms can improve cellular targeting and decrease the systemic off‐target effects when delivering ncRNAs to the infected tissues or to the parasite reservoirs. Even though they show decreased toxicity and increased delivery efficiency in liver‐directed therapeutics in preclinical studies, their use in parasitic diseases is still restricted [26]. Their translational potential is limited by issues including high production costs, limited scalability, and a lack of thorough validation [24, 26].

According to the preclinical studies and strong proof‐of‐concept evidence, LNP‐based delivery methods have proven consistent success in achieving effective gene silencing and immune modulation across multiple disease models [24, 28]. Even though viral vectors have sustained expression and high delivery efficiency, safety concerns limit their clinical use [29]. Ligand‐conjugated systems have better specificity, but they do not have enough extensive validation, especially when it comes to parasite diseases [26].

## 4. miRNA in Diagnosis and Therapeutics in Parasitic Diseases

miRNAs are small ncRNAs that are capable of repressing translation or degrading the target messenger RNAs of parasites. These are important regulators of expression in many biological processes such as cell proliferation, development, differentiation of cells, and the apoptosis of cells [31]. Many studies have found that miRNA can be used as endogenous biomarkers to distinguish between diseased and healthy individuals, to identify the different stages of diseases, and to serve as a tool for screening individuals who are at high risk [7, 32].

In parasitic infections, these miRNAs play an important role in improving host immune responses [33]. One of the primary mechanisms is through the control of cytokine production. Here, specific miRNAs can suppress pro‐inflammatory cytokines like tumor necrosis factor‐alpha (TNF‐*α*) and interleukins. Because of these actions, they reduce excessive inflammation and prevent tissue damage. In contrast, parasites may use host miRNAs to downregulate immune responses, facilitating immune evasion and chronic infections [18].

Besides that, miRNAs influence the differentiation and activation of immune cells, including dendritic cells, macrophages, and T lymphocytes. For example, miR‐155 and miR‐146a are the main regulators of innate immune signaling pathways, such as the Toll‐like receptor (TLR) pathway, which is important in parasitic infections [21]. Malfunctioning of these miRNAs can lead to defective recognition of pathogens or hyperbolic inflammatory responses [18].

Other than that, miRNA‐like molecules can directly interact with host cellular pathways. These molecules can alter expression to create a survivable environment for the parasites. This cross‐species regulatory mechanism emphasizes the complexity in between the host and parasite, which is mediated by miRNAs [19].

### 4.1. Malaria

Malaria can increase the production of inflammatory cytokines such as tumor necrosis factor‐*α* (TNF‐*α*) and interferon‐*γ* (IFN‐*γ*) that can control the parasites but can also lead to severe malaria [34]. Severe malaria can affect the brain, lungs, liver, and kidneys. However, identifying specific molecular markers associated with malaria can decrease its effects. However, people with SCA (sickle cell anemia) can resist malaria parasites because of the potential role of a specific miRNA, known as miR‐451, that can translocate to malaria parasites. But when the miR‐451 in HbSS erythrocytes is inhibited, there are more chances of malaria parasites [34]. According to this finding, miR‐451 plays a major role in inhibiting *Plasmodium falciparum*, which is the parasite of malaria, that is observed in people with SCA [35].

Upregulation of several miRNAs, such as miR‐223, miR‐145, and miR‐155 (Table 2), can be found in patients with *Plasmodium vivax* compared to healthy individuals [39]. As miR‐223 is involved in modulating immune responses by regulating cellular activation, while polarizing the macrophages into M1 and M2 phenotypes, it can suggest the involvement of immune modulation during malaria in *P. vivax*‐infected individuals [34]. MiR‐145 is associated with the reduction of inflammation. Downregulation of miR‐145 can be seen in the context of sepsis, but it has been upregulated in lipopolysaccharide‐induced inflammation and sepsis‐induced lung injuries. This happens through the TGFBR2 signaling pathway, which shows that miR‐145 can play a role in mitigating complications associated with the inflammation during malaria infection [40].

**Table 2:** Regulation of miRNA in different types of parasitic diseases.

Infectious disease	Upregulated miRNA during infection	Downregulated miRNA during infection	References
Malaria	miR‐223 miR‐19b miR‐145 miR‐155 miR‐146a miR‐150 miR‐222 miR‐3158	miR‐451 miR‐16	[34] [36]
Leishmaniasis	miR‐30a miR‐155 miR‐671 miR361 miR‐548d	miR‐193b	[34]
Trypanosomiasis	miR‐193b miR‐338	miR‐199a‐3p miR‐27b miR‐126	[34] [36]
Chagas disease	miR‐34a miR‐208a miR‐185 miR‐223 miR‐454 miR‐512 miR‐515		

Helminthic diseases (Schistosomiasis	Sha‐mir‐71a Sha‐miR‐125b		[37] [38]
(Clonorchiasis)	miR‐16‐2 miR‐93 miR‐95 miR‐153 miR‐195 miR‐199a‐3p	miR‐124a let‐7a let‐7i	

MiR‐155 level is increased among patients who are suffering from cerebral malaria. Infection control, neuroinflammation, and negatively regulating blood–brain barrier integrity and the T cell function are the processes that can be done by miR‐155 [41]. The experimental methods that have been conducted have proven that the pretreatment with miR‐155 inhibitors can help preserve blood–brain barrier integrity [34]. According to these findings, miR‐223, miR‐145, and miR‐155 can play a crucial role in responses related to malaria disease.

According to the study by Gupta and Wassmer [42], children who are suffering from severe malaria reveal higher amounts of miR‐3158 and miR‐4497 when compared to those with uncomplicated malaria. Both show positive association with the histidine‐rich protein 2 expression, which is produced by *P. falciparum* in the blood cycle and can be commonly used as a biomarker for infections. MiR‐3158 can act as a biomarker for cerebral malaria [42]. MiR‐3158 expression levels were associated with the cerebral malaria patients′ mortality, which are linked to the pathway of brain hypoxia. This shows the relevance of miR‐3158 as a prognostic marker and its role in cerebral malaria pathophysiology [34].

All the biomarkers that have been mentioned, such as miR‐223, miR‐145, miR‐155, miR‐3158, and miR‐4497, have therapeutic targets in malaria by modulating immune responses, controlling inflammation, and pathogenesis of diseases.

### 4.2. Leishmaniasis

In the infection of *Leishmania donovani* in humans, researchers have observed an increase in the expression of miR‐30a [34]. MiR‐30 upregulation is associated with the reduction of the load of the parasite, which can be achieved through the autophagy regulation [43]. Those findings revealed that miR‐30 can be used to control the parasite and to combat leishmaniasis.

Using next‐generation sequencing, dysregulation of miRNAs was identified through the in silico analysis of CD4+ T cells with the *L. donovani*‐infected mice macrophages, which have been cultured [34]. They found 11 upregulations and nine downregulations related to the miRNAs with immune response and the dichotomy of T helper 1 cells (Th/1). The transcriptional factors associated with the Th1 phenotype are the ones which are targeted by the upregulated miRNAs, whereas the miRNAs that are downregulated direct cells towards the T helper 2 cells (Th2) population. It indicates that the Th2 response in this infection has a prevalence that is modulated by miRs [44].

Nunes et al. [34] discovered that individuals with cutaneous leishmaniasis (CL), which is brought on by *Leishmania braziliensis*, have miRNA expression in their lesions. They found the miR‐193 b, miR‐671, and the target gene triggering receptor expressed on myeloid cells‐1 (TREM 1), which were related to the healing time [34]. Those three were observed in patients whose lesions healed in the initial treatment cycle, which is up to 59 days after diagnosis, and they can be used as prognostic indicators for CL [45].

### 4.3. Trypanosomiasis and Chagas Disease

This is also known as sleeping sickness; it cannot be controlled by early diagnosis, as they do not show any early‐stage symptoms [36]. But the levels of miRNA such as miRNA‐199a‐3p, miRNA‐27b, and miRNA‐126 are significantly decreased when infected by this parasite compared with the healthy ones. After infection with the *Trypanosoma brucei*, miRNA deregulates the TLR signaling and the signaling of nuclear factor kappa‐light‐chain‐enhancer of activated B cells (NF‐ҡb) [46]. But patients with trypanosomiasis show higher expression levels of miRNA‐193b and miRNA‐338 when compared to the healthy controls. Therefore, biomarkers like miRNA can be used to identify trypanosomiasis and Chagas disease [36, 46].

*Trypanosome cruzi* is the parasite that causes Chagas disease [47]. When studying chronic Chagas cardiomyopathy (CCM) infected patients, scientists identified expression levels of six circulating miRNAs, such as miR‐34a, miR‐208a, miR‐185, miR‐223, let‐7d, and miR‐454. Among all these miRNAs, only miR‐223 acted as the marker of the function of myocardial [34]. miR‐223 could work as a circulating biomarker for heart failure in patients with CCM, because it is remarkably abundant in the left atrium area in the volumes of end‐systole and end‐diastole of the left ventricle [48].

### 4.4. Helminthic Diseases

During *Schistosoma japonicum* infection, host miRNA, known as miR‐223, has been proposed as a biomarker for liver pathology [49]. The levels of miR‐223 increased when the host was infected with the infection and returned to normal values when treated with praziquantel. This was done on a mouse model [50]. In addition to these, changes that occur in host miRNA in infectious diseases can be identified as mechanisms that regulate the induction of protective or suppressive immune responses. In a variety of murine model systems, miRNA has become an important regulator of both innate and adaptive immunity responses [51]. Using a mouse model infected with filariae, it was demonstrated that there was a different expression of a small number of host miRNA when macrophages were activated [52]. In a mouse model infected with *Schistosoma mansoni* infection, regulatory T cells play an important role in reducing pathology, whereas, during a Th2 response, host miRNAs are specifically induced. miRNA‐182 is important in regulating the expression of genes that are needed for stability and expansion of T cells under Th2‐inducing conditions [50]. *Schistosoma haematobium* has miRNA such as Sha‐mir‐1, Sha‐mir‐71a, Sha‐mir‐125b, Sha‐mir‐7a, and Sha‐mir‐let7, which are similar to miRNA in *S. mansoni* and *S. japonicum* [53]. Using host biological fluids, such as serum, can identify schistosomal miRNAs (bantam and miR‐2c‐p3), which can be used to diagnose parasitic organisms and prevent infections. Sha‐mir‐71 can be used as a biomarker in bladder cancers caused by helminthic parasites [54–56].

Sha‐mir‐71a binds to 3 ^′^UTR elements of more than 53 host genes, but it has not yet been experimentally explained. Sha‐mir‐71a can act as a biomarker in bladder cancers, because it has been identified in the urine sample of a patient with bladder cancer [54]. Sha‐mir‐71a was abundantly found in the urine sample of patients with bladder cancer compared to patients with benign bladder cystitis associated with schistomiasis [55]. As Sha‐mir‐71a expression varies among different types of cancers, for example, traditional cell carcinoma and squamous cell carcinoma, Sha‐mir‐71a can be used as a biomarker to detect cancers caused by helminthic parasites [54].

## 5. miRNA in the Therapeutics of Parasitic Diseases

### 5.1. Leishmaniasis

Few studies on leishmaniasis have found the miRNA profile during the major infection that occurs in human macrophages. In this case, host miRNAs can affect macrophage activation and parasite survival, whereas parasite ncRNAs support gene expression programs necessary for persistence and transmission. This distinction is important for treatment design, since ncRNA‐based interventions can improve disease control by targeting host regulatory networks or parasite‐specific pathways [18]. One of them identified 64 miRNAs with significant modulations [34]. MiR‐210 shows increased expression in a HIF‐1*α*–dependent manner and can be seen in responses to stress and signal transduction [57]. After 24 and 48‐h postinfection in macrophages exposed to leishmaniasis, let‐7a was found to be downregulated. This downregulation is associated with increased cellular apoptosis and necrosis, which leads to the control of infection [58]. When miR‐340 expression decreases, it can promote parasite survival. Immune regulatory cytokines such as interleukin‐10 (IL‐10) and transforming growth factor beta‐1 (TGF‐*β*1) are the targets that show increased expression due to miR‐340 knockdown. When transfection of infected macrophages with miR‐340 can decrease the macrophage infectivity, this suggests that miR‐340 can be used as a therapeutic agent in the treatment of CL [59, 60].

These explanations clarify the interplay between cytokines and miRNAs in the regulation of the host immune response by activating the inflammasome during CL [61]. According to these studies, the expression levels of miR‐7‐5p, miR‐133a, miR‐146b, miR‐223‐3p, and miR‐328‐3p, as well as the levels of cytokines such as IL‐1*β*, IL‐6, and IL‐17, were increased in patients with CL compared to the healthy ones. Observing these points suggests that these molecules help in the diagnosis, prognosis, and treatments of leishmaniasis [34].

### 5.2. Trypanosomiasis and Chagas Disease

There is an upregulation of miR‐145 and miR‐146b in infected cardiomyoblasts related to *T. cruzi* infection, which may help host defense mechanisms by increasing immune responses or by altering the parasite′s life cycle [62]. Due to upregulation of the abovementioned miRNA, it can influence cellular pathways such as cellular proliferation, apoptosis, and inflammation. Examples of such cellular pathways are miR146b, which can be used in the regulation of the response to immunity and inflammation, which helps to control the inflammatory activities associated with *T. cruzi* infection [34]. Regulating miR‐145 and miR‐146 b levels can be used to improve host immunity and decrease parasite load [62].

In Chagas disease, placenta‐specific miRNAs such as miR‐512 and miR‐515 are mainly important. By inhibiting cellular FLICE‐like inhibitory protein (c‐FLIP), miR‐512 promotes trophoblast differentiation, and miR‐515 inhibits differentiation by upregulation of the human chorionic gonadotropin modulator‐1 (hGCM‐1) [34]. The increase in the level of miR‐512 and the decrease in the level of miR‐515 indicate the placental defense mechanism against the *T. cruzi* parasite in trypanosomiasis. These miRNAs are most important in trophoblast turnover and defense response in *T. cruzi* infection [38].

### 5.3. Helminthic Diseases

Some miRNAs may regulate immune responses during an infection with helminths. Expressions are shown by miR‐142a, miR‐181a, and miR‐223 in immune cells [63]. As many of the miRNAs are used in proliferation, regulation of maturation, differentiation, and activation of immune cells, miR‐17‐92 cluster, miR‐150, miR‐155, miR‐181, miR‐206, miR‐223, and miR‐511 are highly expressed in immune cells such as macrophages, B cells, T cells, and monocytes which are directly participating in the development of cells and immune regulation [64].

During schistosomiasis, miRNAs, known as miR‐155, miR‐223, and miR‐146, are known to be suppressors of the TLR and cytokine signaling via a negative feedback regulation loop. This loop is involved in the downregulation of IL‐1 receptor‐associated kinase 1 (IRAK1) and the TNF receptor, which is associated with factor 6 (TRAF6) protein levels [65]. MiR‐155 is associated with the c‐Maf transcription factor and also helps in the Th2 response in CD4+ T cells [66]. According to a study of mice that were infected with Schistosoma, these miRNAs are primarily associated with immune regulation, nutrient metabolism, cell proliferation, and molecular regulation and differentiation [37]. miR‐146a and miR‐146b control macrophage differentiation to M2 cells, which shows excessive processes in inflammation. Not only that, they also secrete cytokines such as IL‐10 and TGF‐*β* to promote protective responses [67]. IL‐10 plays immunosuppressive roles in helminthic infections, and also the TGF‐*β* promotes tissue fibrosis through the process of overproduction of Type I collagen [38].

In Clonorchiasis, Csi‐let‐7a‐5p, located inside the macrophages, binds to the 3 ^′^UTR (untranslated regions) of Socs1 (a cytokine signaling suppressor) and Cle7a (C‐type lectin domain consisting of 7a), which regulate the decrease in mRNA and protein expression [55]. Because of this mechanism, it activates the NF‐ҡb signaling pathway. Csi‐let‐7a‐5p drives macrophage polarization toward the M1‐like phenotype by upregulating key proinflammatory cytokines (TNF‐α, IL‐6, and IL‐1β), inducible nitric oxide synthase (NOS2), and the surface activation markers CD80 and CD86. These molecular changes reflect the acquisition of a classically activated inflammatory phenotype associated with heightened immune effector responses [32]. Due to this proinflammatory effect, it contributes to the induction of biliary injury, and this Csi‐let‐71‐5p is important in carcinogenesis and can be a target for the therapy or prevention of Clonorchiasis [55].

Although miRNA‐based treatments are moving closer to clinical use, they are still not as developed as siRNA‐based strategies. The specificity, safety, and predictability of therapeutic effects are challenged by the complexity of miRNA‐based regulations, including the ability to target multiple mRNAs at once and affect entire gene networks [18]. Although preclinical research has shown that miRNAs can affect infection outcomes and host immune responses, clinical translation demands validation to reduce off‐target effects and guaranty regulation. With an estimated 5–10 years of clinical application in parasitic infections, miRNA‐based therapies are therefore expected to follow a similar but slightly longer timeline than siRNA‐based therapies [24, 68, 69].

## 6. siRNA Used for Therapeutics in Parasitic Diseases

By Dicer ribonuclease, siRNA is formed from endogenous or exogenous long dsRNA. From exogenous or endogenous long dsRNAs by using Dicer ribonuclease, the siRNAs are produced. To identify and cleave the sequence of homologous mRNA, the primary siRNAs interact with the RNAi‐defective protein‐1 (RDE‐1) Argonaute protein. Secondly, siRNA amplifies the response produced by RNA‐directed RNA polymerase (RRF‐1) [70]. Key mediators of RNAi (RNAi‐mediated gene silencing) are siRNA [71]. In parasitic diseases, siRNAs play an important role in regulating immune responses by selectively targeting and degrading mRNAs that are involved in infection and inflammation [72]. These siRNAs silence genes that are associated with parasite survival and host immune evasion. SiRNA can inhibit essential biological processes like metabolism, replication, and development by targeting parasite‐specific genes, which reduces the viability of parasites [24].

According to some studies, RNAi is used in gene silencing in parasitic helminths, which helps to detect and activate a functional pathway of silencing. To obtain the needed functional genomic tool, the mechanism of silencing the gene of interest should be performed [50]. As siRNA‐based treatments are more specific for gene silencing, this makes them particularly relevant for targeting parasite‐specific pathways with high precision.

In siRNA‐mediated immune responses, siRNA‐mediated suppression of host genes involved in inflammatory pathways can reduce excessive immune activation and tissue damage. In contrast, siRNAs can enhance immune responses by silencing negative regulators of immunity, enhancing the removal of pathogens [19].

### 6.1. Cystic Echinococcosis (CE)

This is a helminthic disease that is caused by a parasite called *Echinococcus granulosus*. In all eukaryotic organisms, calmodulin (CaM), which is a multifunctional intermediate calcium sensor protein, is expressed [73]. CaM is used on many occasions, such as Ca^2+^ binding and helping in the calcium signal transduction pathway to help regulation of biological processes such as reorganization, activation of phosphorylase kinase, responses to abiotic stress, neurotransmission, smooth muscle contraction, metabolism, and motility of cells [74]. To obtain more information about the developmental biology of Platyhelminthes, siRNAs play a major role as a valuable tool [75]. RNAi‐based molecular methods and post‐transcriptional gene silencing (PTGS) are tools that provide information on gene function, and gene characterization can be used in regeneration and development. siRNA, which is a type of RNAi, is used to cause gene silencing for experimental and therapeutic purposes [76, 77]. RNAi technology is used mainly to suppress CaM in *E. granulosus.* According to the new experiments, siRNA sequences are used for the suppression of EgCaM (*E. granulosus* CaM) to identify different stages of development of the above parasite [73]. Different siRNA produce suppression at the mRNA level. Ninety‐two percent of the suppression was observed through the electro‐soaking in all developmental stages. Because of this, electroporation produces some pores in membranes, and it allows the entry of siRNA in the transfection buffer [78]. There is robust suppression in EgCaM gene expression, which causes the changes in phenotype, viability, and inhibition of growth in protoscoleces that are treated by siRNA [73].

Chapman and Carrington [79] successfully suppressed gene expression treated with 14‐3‐3 and *elp* siRNA by 21.8% and 35.5%. On Day 15, according to the viability, 14‐3‐3 and *elp* siRNA‐treated samples showed 58.0*%* ± 23.0*%* and 55.1*%* ± 14.6*%*, respectively, when compared to the control sample, which was untreated. Further research findings revealed that EgCaM‐specific siRNA in the protoscoleces induces the growth and inhibits the transformation of protoscoleces into microcysts compared to the controls. So, it shows that the reduction of CaM expression can prevent the growth and development of the helminths [2].

Due to that, it causes shrinkage of the outer walls of the microcysts [80]. EgCaM expression suppression was observed in the cells after 8 days of siRNA treatment. Also, from the treated worms, waves of contraction or dilation were observed [78]. Furthermore, siRNA‐mediated decrease of 24lDa calcium‐regulated heat‐stable protein (CRHSP‐24) in juvenile parasites may cause death or can change the morphology of the parasite [70]. The suppression of EgCaM in the *E. granulosus* strobilated forms leads to a decrease in motility and contractions of the strobilus, whereas the untreated control and negative siRNA groups were active and showed normal contractions [81]. CaM, mitogen‐activated protein kinase (MAPK), and Polo‐like kinase (PLK), which are types of Ca^2+^‐dependent protein kinases, are playing a major role in the motility of invertebrates [73, 78]. It shows a significant increase in the motility, migration, and movement of juveniles of parasites because of the RNAi and CaM inhibitors.

It is evident from the findings gained from many studies that the effect of siRNA for the suppression of EgCaM in different life stages of *E. granulosus* was successful. This EgCaM is very important in the viability, growth, and development of the protoscoleces. These phenotypic changes may be used as therapeutic agents against CE [73].

### 6.2. Scabies Disease

One of the most common parasitic skin infections is caused by *Sarcoptes scabiei* [82]. In Scabies mite in vitro experiments, RNAi has become an important experimental approach to test potential drugs and vaccines [83]. This gene silencing is done by means of RNAi, which is triggered by dsRNA. This dsRNA is introduced into the mites by feeding, and then after dsRNAs enter cells, they may induce systemic RNAi effects [84].

Enzymes named GST are important for the detoxification of insecticides within insects [85]. In recent studies, they have used *ssGST-mu 1* as the first target in *S. scabiei* for gene silencing, as it was the gene that has been studied up to the transcriptional level so far [73]. In honeybee mites, GSTs can be found to be equally and constitutively expressed in the synganglion, Malpighian tubules, and in the gut, whereas GSTs are housekeeping genes [86]. *S. scabiei* consists of at least six glutathione S‐transferase genes [86]. Three of those genes are in GSTs of the mu class, and the remaining three are in delta/epsilon classes that are of special interest in the assessment of drug resistance [87]. In many research studies, *GST-mul* localized in the gut *S. scabiei* is used because it is more dominant in mite bodies [88]. After removal of the parasite from the host, SsGST showed increased expression [85]. According to optimization of *SsGAT-mul* and *Lac*Z‐dsRNA as control, these researchers have applied RNAi to do gene silencing for drug targets such as SsSar s 3 and SsAP [77, 89].

### 6.3. Cryptosporidiosis

This is a parasitic disease that causes diarrhea primarily in children, which is caused by the infection of *Cryptosporidium parvum*. The silencing of genes in *Cryptosporidium* with the use of single‐stranded RNA (ssRNA) or Argonaut 2 (Ago) complexes indicated the elimination of the expression of nucleoside diphosphate kinase (NDK), which can block the proliferation of the parasite. As *Cryptosporidium* lacks enzymes in the RNA‐induced silencing complex (RISC), the silencing process is based on the siRNA reconstitution pathway [19]. According to past studies, they have shown that the passage of nucleic acids through oral methods is not efficient [90]. Because of that, in recent studies, researchers found a method using a system named a cationic lipid‐based system. In this process, liposome is attached to the negatively charged surface and can freely fuse through the membrane. As *Cryptosporidium* does not have caveolins, ssRNA is entered into the membrane by clathrin‐mediated endocytosis [91]. According to previous studies, they have shown that NDK silencing blocks parasite proliferation without cytotoxic effects [91]. In vitro experimental results showed that parasite proliferation was reduced in infected cells and daily treatment increases anticryptosporidial activities. siRNA, which is encapsulated in lipidic nanoparticles, is effectively moved through the oral cavity into the mouse intestines. ssRNA was also delivered in vivo, the same as siRNA, using the cationic lipids used in the in vitro experiments [92].

According to in vitro and in vivo experimental results, they showed that the anticryptosporidial effects of NDKs ssRNA complexes may affect the inhibition of many biological functions in which NDKs are major [91]. The most important role of NDK is in the synthesis of nucleotides. However, in *Cryptosporidium*, they do not need purine nucleotide synthesis according to previous studies. Inhibition of NDK may affect other functions of *Cryptosporidium*; for example, this is used to reduce immunity, apoptosis, and inflammation. In *Propionibacterium gingivalis*, NDK inhibits the extracellular ATP (eATP), which mediates cell death in epithelial cells through eATP hydrolysis [93]. In Leishmaniasis, NDK also helps evade the immune system by preventing the death of macrophages mediated by eATP [94]. In order to prevent host cell death, secreted NDK kept the membrane of the host cell intact and stabilized the mitochondrial membrane potential in macrophages [95]. According to the studies, they showed that the usage of RNAi, especially siRNA as a drug or therapeutic method, is used to control the activities of the *Cryptosporidium* parasite.

In terms of clinical translation, siRNA‐based therapies are the most advanced class of ncRNA interventions. In nonparasitic diseases, strong evidence for the clinical viability of siRNA‐based treatments is provided by their successful development and regulatory approval, particularly using the LNP delivery method [24, 26]. According to current clinical success in other diseases, a realistic timeline of roughly 5–10 years is required to adapt and translate into parasitic disease therapeutics, although siRNA applications in parasitic diseases are still mostly in the preclinical stage [24].

## 7. Long ncRNA Used in Diagnosis and Therapeutics in Parasitic Diseases

According to experiments, lncRNAs are used in the innate and adaptive immune system [56]. These lncRNAs are involved as regulatory nodes for activation and help increase the immune signals, as transcriptional factors, and for infection coregulators and co‐activators for genes related to immunity [56, 80]. In addition to that, lncRNAs help in pro‐inflammatory and anti‐inflammatory responses, differentiation in immune cells, and secretion or inhibition of cytokines [56]. The transcriptional regulation of intestinal epithelial cell (IEC) defense genes is functionally induced by the specific lncRNA in the infection of *C. parvum* [96]. After infection with C. *parvum* NR‐045064, transcriptional regulation in host cells, similar to that observed during *Toxoplasma gondii* infection, may involve lncRNAs that negatively regulate UNC93B1 expression and secrete pro‐inflammatory cytokines [53]. Although their roles in parasitic diseases remain less specific, emerging evidence shows that lincRNA‐based therapies are more involved in regulating host immune responses and potentially mediating host–parasite communication.

In order to activate inflammatory reactions, during *Eimeria necatrix* infection, lncRNA can downregulate host defense genes by using recruitment of TLRs or by inducing phosphorylation [14]. In addition, in *T. gondii* infection, lncRNAs are involved in macrophage differentiation, cytokine–receptor interaction, JAK‐STAT, and the signaling pathway of p53 [56]. In some parasitic infections, MAPK is used, and lncRNAs are also used as regulators of inflammatory processes in leukocytes [97]. The gene expression of lncRNA is similar to the activation of the NF‐ҡB. More explanations are needed for the NF‐*κ*B–mediated potential transcription and translation [53].

According to some previous evidence, the increase in the expression of myocardial infarction associated transcript (MIAT) in Chagas disease was associated with endothelial cell dysfunction in chronic cardiomyopathy. Due to the expressions of MIAT in *T. cruzi*, it confirms that MIAT is a biomarker for chagasic cardiomyopathy [96]. When infected with *T. gondii*, sival‐205 and nfkbl‐210 show higher expression in myDD8, which is a wild type, than Myd88^−/−^ macrophages [56]. Because of that, lncRNA can act as a biomarker for toxoplasmosis [96]. As lncRNAs can be seen in extracellular vesicles (EV), these molecules can be used as biomarkers to identify parasitic diseases. There is a possibility that recipient cells respond to drugs via EVs that are enclosed in lncRNAs.

However, lincRNA‐based treatments are in the early stages of development. Although they have been linked to host–pathogen interactions, chromatin remodeling, and gene regulation, their functional role in parasitic diseases is still unclear [17]. Challenges related to large molecular size, complex structural properties, and lack of efficient delivery systems are some of the problems with the treatment of lincRNA‐based therapies. Instead of providing a functional intervention, the majority of current research is more focused and concentrated on finding patterns of lincRNA expression [14, 98]. Because of that, lincRNA‐based treatments will take longer than 10 years and will require substantial advancements in both delivery technology and mechanistic understanding [17].

## 8. Enhanced Comparative Analysis of ncRNA‐Based Therapies and Conventional Antiparasitic Treatments

### 8.1. ncRNA‐Based Versus Conventional Therapies: Comparative Risk Profile

Conventional antiparasitic and ncRNA‐based therapies have different risk profiles based on clinical maturity, predictability, and mechanisms. Clinical trials and long‐term usage of conventional medicines have a well‐documented safety profile. However, their disadvantages are becoming more evident, especially the development of drug resistance, decreased effectiveness against tissue‐resident or dormant parasite stages, and accumulation of the toxin form in long‐term therapies [99].

On the other hand, ncRNA‐based treatments are still in the experimental and preclinical phases, where pharmacokinetics, biodistribution, and long‐term safety are unpredictable. Unintentional gene silencing, immunological activation, circulatory instability, and difficulties attaining targeted distribution to infected tissues are some major damages that can occur. Furthermore, because ncRNAs are pleiotropic and have a cascade effect, changing one of them can affect several cellular pathways, making safety evaluations more difficult [24].

Even though there are a few concerns about ncRNAs therapies, they offer several advantages compared to conventional drugs. These ncRNA treatments can be used to treat target genes that cannot be accessed by conventional drugs, can simultaneously regulate multiple pathways that are involved with infections and immunity, and can provide host‐directed therapies that can decrease the parasite′s survival without directly targeting the parasite [100].

Therefore, ncRNA‐based therapies have less predictability but possibly more precise and adaptive dangers, which can be optimized by advancements in delivery systems and molecular designs.

### 8.2. Comparing ncRNA‐Based Therapies to Conventional Treatments: Off‐Target Risks

The possibility of off‐target effects due to complicated regulatory networks and poor sequence specificity is a major drawback of ncRNA‐based treatments. Because of the default nature of miRNAs, they can bind to multiple mRNA targets through partial complementarity; other than single genes, miRNAs can regulate entire gene networks [24]. Although this characteristic is beneficial for extensive regulatory control, it increases the possibility of unintended host cellular pathways, while increasing the chances of immune response dysregulation [16]. Despite their high specificity, siRNAs can activate the innate immunity pathways such as TLRs and interferon responses, while causing off‐target gene silencing through partial sequence homology or “seed region” interactions [24, 101].

Conventional antiparasitic medications typically work within the parasite through specific biochemical processes. For example, benzimidazoles disrupt helminth microtubule polymerization, and artemisinin‐based treatments produce reactive oxygen species that harm parasite proteins. Although these pathways are not entirely parasite specific, they are relatively well characterized, and their adverse effects are related to systemic toxicity [88]. Most importantly, these conventional drugs do not usually interact with host gene expressions at a regulatory level, which reduces the risk of widespread molecular perturbations.

Even though ncRNA‐based therapeutics introduce network‐level off‐target risks, their potential for increased specificity still depends on further optimization and validations [24]. On the other hand, conventional treatments are linked to well‐defined, dose‐dependent toxicity. Because of this, both treatments provide unique safety issues that have to be evaluated in the context of parasitic disease treatments.

## 9. Limitations of Current Evidences and Challenges Related to ncRNA‐Based Therapies

Previous studies on ncRNA have shown that their use as biomarkers and therapeutic tools for parasitic diseases has been a success. However, there are some challenges facing ncRNA in therapeutics on specificity, delivery, and tolerability [56].

These ncRNAs are used in processes such as immune cell development and functions, immune disorders, and neural development [97, 100]. The main challenge in this is a limited understanding of ncRNA in parasitic diseases. Significant knowledge gaps exist regarding the role of ncRNA in disease pathology, diagnosis, and therapy [102]. Although miRNAs have been studied in malaria, the effect of lncRNA in tropical diseases like African trypanosomiasis has been neglected [103]. There are some challenges regarding the delivery mechanisms; it is important to deliver the ncRNA therapeutics to the infected tissues. The successful application of ncRNA‐based therapeutics mainly depends on the training of healthcare professionals. In contrast to traditional treatments, ncRNA therapies may require specific delivery systems and close observation for immunological reactions and off‐target consequences [24, 26]. However, with the knowledge gained from the current studies, some issues faced with the delivery, such as circulation instability, nuclease degradation, and the lack of specificity, may lead to off‐target effects [96]. Therefore, the need for focused training and capacity‐building programs is highlighted by LMICs′ limited access to qualified workers [99].

The off‐target effects and safety of ncRNAs are affected by the interactions of ncRNA with multiple mRNAs, which can increase the risk of unplanned gene regulation [102]. Another challenge is that there are regulatory and ethical considerations; there can be many ethical concerns when conducting gene expression, studies especially in human trials [103].

The unequal distribution of ncRNA research in the parasitic diseases shows a larger difference in funding allocations, research infrastructure, and global health goals (GHGs). Advanced infrastructure, including RNA synthesis, purification, and formulation, is necessary for the large‐ scale manufacture of ncRNA therapies [24, 26]. Furthermore, methods such as LNP formulation are crucial for preserving stability and effectiveness as they require cold‐chain storage [28]. ncRNA‐based therapies continue to be costlier than traditional treatments due to expenses related to these processes [28]. These requirements create significant challenges for LMICs, where infrastructure and technical competence are limited [99]. Therefore, malaria‐like high global burden diseases have received substantial attention, whereas many neglected tropical diseases (NTDs), such as African trypanosomiasis, schistosomiasis, and filariasis, remain relatively understudied [104]. The reasons for the constraints of ncRNA research in endemic regions may be limited access to advanced molecular technologies, including next‐generation sequencing and bioinformatics tools. Moreover, ncRNA discovery and functional validation are significantly challenged by complex life cycles, genetic diversity, and host–parasite interactions [19]. Together, these factors contribute to the existing knowledge gap and impede the advancement of ncRNA‐based therapeutic and diagnostic applications.

Apart from these, a crucial inquiry is whether the results from well‐researched diseases, especially malaria, can be applied to less‐researched parasitic infections. Research on malaria has provided valuable insights into the role of ncRNAs in regulating immunity, inflammation, and host–parasite interactions, particularly through the modulation of cytokine signaling and gene expression pathways [18]. However, the applicability of these findings has been limited because of the biological differences among the parasites. Differences in life cycles, tissue tropism, intracellular lifecycle versus extracellular lifecycles, and immune evasion strategies greatly influence ncRNA expression and function. Extracellular parasites such as *T. brucei* use different bloodstream survival strategies, whereas intracellular parasites such as *Leishmania* spp. interact closely with host macrophages. Additionally, the direct transferability of regulatory mechanisms found in one system to another is limited by the low conservation of ncRNA targets across parasite species [19]. Therefore, species‐specific validation is necessary for appropriate interpretation and use in other parasite diseases, even though malaria, like well‐studied diseases, offers a useful conceptual foundation.

To address these challenges and limitations, target funding for ncRNA research in neglected diseases must be increased to promote the balanced scientific advancements [99, 104]. The identification and comparative investigation of ncRNAs across species will be made easier by expanding genomic and transcriptome resources for under‐represented parasites, especially with the development of integrated omics techniques and high‐throughput sequencing technology [88, 105].

Collaborative research networks are especially important in this situation because they allow the sharing of data, infrastructure, and expertise.

## 10. Strategies to Improve Safety, Specificity, and Translational Feasibility of ncRNA‐Based Therapeutics

A hybrid delivery system is a mechanism that can be used to increase delivery efficiency and specificity. LNPs combined with targeting ligands or biomimetic coatings can be considered. These strategies aim to decrease systemic toxicity and off‐target distribution while increasing cellular uptake [25, 26]. Combining the precision of targeted systems with the scalability of lipid‐based distribution may offer a well‐rounded solution, making them better options for further therapeutic research.

Computational approaches are essential for improving the specificity of ncRNA treatments by anticipating possible off‐target interactions. Unintentional binding sites can be found using bioinformatics tools and machine learning (ML)‐based algorithms, especially for miRNAs and siRNAs, which frequently show partial sequence complementarity [24]. For example, miRNA–mRNA interactions based on seed region complementarity and thermodynamic stability are frequently predicted using algorithms such as TargetScan, miRanda, and RNAhybrid [87, 106]. By combining sequence characteristics and experimental datasets, ML‐based methods have been providing more accurate details. These computational techniques can improve therapies related to ncRNA by optimizing ncRNA design and reducing unwanted gene regulations [24].

Chemical modifications of ncRNAs are most commonly used to improve stability, specificity, and resistance to nuclease destruction for therapeutic and diagnostic purposes. In leishmaniasis and malaria, to enable the in vivo applications, nuclease resistance and binding affinity are enhanced by 2 ^′^‐O‐methyl (2 ^′^‐O‐me) and 2 ^′^‐fluoro (2 ^′^‐F) substitutions to enable in vivo application [24, 107]. Locked nucleic acids (LNAs) are especially helpful in identifying parasite‐specific miRNAs in diagnostic assays and further improve hybridization stability [69]. New approaches, such as GaINAC conjugation, offer targeted delivery potential; however, their use in parasitic diseases is still limited [24].

The clinical translation of ncRNA therapies depends on the discovery of safe and efficient dosage schedules. Preclinical screening techniques such as dose–response analyses, transcriptome‐wide off‐target profiling, and immunogenicity evaluations have been used to identify the therapeutic windows and reduce toxicity [26]. These approaches make dosage optimization possible while guaranteeing safety and effectiveness in later clinical studies.

Specialized regulatory frameworks that consider the distinct mechanisms of action of ncRNA‐based therapies are necessary for their development. Regulatory evaluation must consider factors such as off‐target gene regulation, long‐term safety, and interactions in the delivery system [24]. For example, standards for oligonucleotide therapies have been published by regulatory bodies, including the European Medicine Agency (EMA) and the US Food and Drug Administration (FDA). These guidelines include requirements for long‐term toxicity research, immunogenicity testing, and preclinical safety evaluations. Frameworks for approved siRNA‐based medications, such as patisiran, highlight the assessment of pharmacokinetics, gene‐silencing specificity, and LNP delivery techniques [26]. Constantly, these regulatory frameworks are changing as RNA‐based therapies are still relatively new; because of that, health authorities must work together to standardize evaluation criteria to guarantee safe clinical translation.

In addition to regulatory considerations, the success of ncRNA therapies is also influenced by the economic factors. Variations in healthcare infrastructure, disease burden, and affordability can differ according to the economic situation. Since LMICs prioritize cost‐effective mass drug administration tactics from the standpoint of the public health system, ncRNA treatments would now only be justified in focused or outbreak‐specific interventions rather than universal use. To achieve global fairness in implementation, the cost–benefit balance shifts from individual‐level precision benefits in HICs to population‐level affordability limits in LMICs. This underscores the need for simple, scalable, and locally adaptive ncRNA delivery systems [26, 28].

## 11. Pursuing ncRNA‐Based Methods Despite Current Limitations

The substantial and expanding limits of traditional antiparasitic treatments justify the ongoing investigation of ncRNA‐based therapeutics. Drug resistance has become a serious global concern, especially in leishmaniasis and malaria, where parasites have developed strategies to avoid pharmacological interventions, including drug efflux, target alteration, and metabolic adaptability [99]. In addition, many current drugs have stage‐specific activity, which limits their efficacy against parasites with complicated life cycles, such as latent or intracellular stages [81]. Because of the toxicity and the long‐term treatments associated with some antiparasitic drugs, treatment success and patient compliance are lower.

Treatments based on ncRNA target gene expression rather than biochemical mechanisms. For example, siRNA‐based silencing of essential parasite genes can directly inhibit parasite survival, whereas miRNA‐based control of host immune responses can improve pathogen clearance and lower disease severity [18, 72, 76]. Compared with conventional therapies, which mainly focused on parasite‐specific pathways, ncRNA‐based dual targeting on both host and parasite is a major advantage.

Furthermore, ncRNAs show potential for applications in precision medicine, where patient‐specific gene expression profiles can guide targeted interventions. Circulating biomarkers also provide chances for early diagnosis, allowing for timely treatment and better disease outcomes [63]. Crucially, these ncRNA‐based therapeutics can be used with existing drugs to increase efficacy, reduce drug resistance development, and lower drug dosages [24].

Another reason for the use of ncRNA‐based treatments over conventional treatments is that conventional in vitro or in vivo models frequently fail to capture the complex regulatory networks involved in host–parasite interactions, which are extremely dynamic and include immunological regulation and epigenetic changes. Many of the current experimental systems, which rely on single‐stage parasite culture or simplified host settings, cannot replicate the temporal and spatial complexity of infection processes. By enabling fine‐scale regulation of gene expression networks in both the host and the parasite, ncRNA‐based therapies provide a distinct benefit by offering a more accurate tool to examine and modify these relationships [24].

In *P. falciparum* malaria, immune evasion and host erythrocyte remodeling are closely related to parasite survival. Both host and parasite gene expression networks control these processes, which can be impacted by miRNAs and siRNAs that target important regulatory pathways [108]. Similarly, host‐derived miRNAs such as miR‐155 and miR‐21 govern parasite survival and immune response outcomes in *Leishmania* infections, where host macrophage polarization is regulated by intricate signaling networks [109].

Despite their importance, ncRNA‐based strategies are still not ready to replace conventional treatments. Rather, they should be used as next‐generation supplementary methods that broaden the range of therapeutic tools available for parasitic diseases [24]. Therefore, more research is necessary to address the present issues of delivery efficiency, off‐target effects, stability, cost, and scalability, especially in places with limited resources where parasitic diseases are more common [24, 99].

## 12. Conclusions and the Way Forward

ncRNAs are RNAS that are not translated into protein but play many functional roles in cells. miRNAs and siRNAs are the most important ncRNAs as diagnostic and therapeutic tools for parasitic diseases. lncRNA plays an important role as a therapeutic tool for parasitic diseases. Under many parasitic diseases, such as malaria, leishmaniasis, Chang disease, and trypanosomiasis, miRNA shows upregulation and downregulation as a biomarker. Furthermore, miRNA can act as a therapeutic tool to diagnose or treat such parasitic diseases. In addition to miRNA, siRNA also plays a huge role in the therapeutic side of parasitic diseases, as they have special mechanisms such as RNAi mechanism of action. In this context, delivery of siRNA to the host body can destroy the parasites from the host cells through RNA‐induced silencing, leading to the degradation of target mRNA. In addition, these siRNAs target specific parasite genes, increase immunity, and act as a vaccination tool against parasitic diseases. lncRNAs also have many successful mechanisms to treat parasitic diseases, and they can act as biomarkers to identify those parasitic diseases. Due to their ability to precisely silence genes, siRNA‐based therapies offer the strongest functional proof‐of‐concept among ncRNA types. Although miRNA‐based methods are more widespread, they show less focused immunomodulatory effects. Conversely, lincRNA‐based approaches have limited functional conformation in preclinical animals and are still primarily exploratory.

In this review, we discuss challenges, such as delivery limitations, low stability, short half‐life, immunogenicity and toxicity issues, off‐target effects, reduced specificity, and ethical and regulatory considerations. With the progression of the research, ncRNA‐based treatments, which are highly specific, minimally invasive, and effective for diagnosis and therapy, may minimize parasitic diseases in the world.

## Author Contributions

K.R.: conceptualization. N.L.G.: writing – original draft, data curation. K.R.: writing – review and editing.

## Funding

No funding was received for this manuscript.

## Disclosure

All authors contributed to the article and approved the submitted version.

## Ethics Statement

The authors have nothing to report.

## Conflicts of Interest

The authors declare no conflicts of interest.

## Data Availability

The data that support the findings of this study are available from the corresponding author upon reasonable request.
